# Correlation between Tissue Cellularity and Metabolism Represented by Diffusion-Weighted Imaging (DWI) and 18F-FDG PET/MRI in Head and Neck Cancer (HNC)

**DOI:** 10.3390/cancers14030847

**Published:** 2022-02-08

**Authors:** Omar Freihat, Tóth Zoltán, Tamas Pinter, András Kedves, Dávid Sipos, Imre Repa, Árpád Kovács, Cselik Zsolt

**Affiliations:** 1 Department of Medical Imaging, Faculty of Health Sciences, University of Pécs, 7621 Pécs, Hungary; david.sipos@etk.pte.hu; 2 Doctoral School of Health Sciences, University of Pécs, 7621 Pécs, Hungary; toth.zoltan@sic.medicopus.hu (T.Z.); kedvesandras94@gmail.com (A.K.); repa.imre@sic.medicopus.hu (I.R.); cselik.zsolt@sic.medicopus.hu (C.Z.); 3 MEDICOPUS Healthcare Provider and Public Nonprofit Ltd., Somogy County Moritz Kaposi Teaching Hospital, 7400 Kaposvár, Hungary; 4 Dr. József Baka Diagnostic, Radiation Oncology, Research and Teaching Center, “Moritz Kaposi” Teaching Hospital, 7400 Kaposvár, Hungary; pinter.tamas@sic.medicopus.hu; 5 Institute of Information Technology and Electrical Technology, Faculty of Engineering and Information Technology, University of Pécs, 7621 Pécs, Hungary; 6 Department of Oncoradiology, Faculty of Medicine, University of Debrecen, 4032 Debrecen, Hungary; 7 Csolnoky Ferenc County Hospital, 8200 Veszprém, Hungary

**Keywords:** diffusion-weighted imaging, apparent diffusion coefficient, head and neck squamous cell carcinoma, PET/MRI, glucose metabolism, primary tumor

## Abstract

**Simple Summary:**

We report on the correlation between the diffusion-weighted imaging (DWI) and the metabolic volume parameters derived from a PET scan, to determine the correlation between these parameters and the tumor cellularity in head and neck primary tumors. Our findings implied that there was no correlation between the information derived from the DWI and the information derived from the FDG metabolic parameters. Thus, both imaging techniques might play a complementary role in HNC diagnosis and assessment. This is significant because the treatment plan of patients with HNC should be well evaluated by using all the available diagnosis techniques, for a better understanding of how the tumor will react.

**Abstract:**

Background: This study aimed to assess the association of 18F-Fluorodeoxyglucose positron-emission-tomography (18F-FDG/PET) and DWI imaging parameters from a primary tumor and their correlations with clinicopathological factors. Methods: We retrospectively analyzed primary tumors in 71 patients with proven HNC. Primary tumor radiological parameters: DWI and FDG, as well as pathological characteristics were analyzed. Spearman correlation coefficient was used to assess the correlation between DWI and FDG parameters, ANOVA or Kruskal–Wallis, independent sample t-test, Mann–Whitney test, and multiple regression were performed on the clinicopathological features that may affect the 18F- FDG and apparent-diffusion coefficient (ADC) of the tumor. Results: No significant correlations were observed between DWI and any of the 18F-FDG parameters (*p* > 0.05). SUVmax correlated with N-stages (*p* = 0.023), TLG and MTV correlated with T-stages (*p* = 0.006 and *p* = 0.001), and ADC correlated with tumor grades (*p* = 0.05). SUVmax was able to differentiate between N+ and N− groups (*p* = 0.004). Conclusions: Our results revealed a non-significant correlation between the FDG-PET and ADC-MR parameters. FDG-PET-based glucose metabolic and DWI-MR-derived cellularity data may represent different biological aspects of HNC.

## 1. Introduction

Head and neck cancer is the sixth most frequent cancer worldwide, accounting for around 6% of all cancer diagnoses and approximately 1–2% of all cancer fatalities [1]. HNC cancers are a diverse collection of malignancies that are anatomically similar but differ in their origin, histology, diagnostic, and therapy techniques [2]. Squamous cell carcinomas account for 91% of all HNC cancers, sarcomas for 2%, and adenocarcinomas, melanomas, and unspecified tumors for the remaining 7% [3].

Recently, ^18^F-fluorodeoxyglucose (FDG) positron emission tomography (PET)/magnetic resonance imaging (MRI) has emerged as an effective and accurate imaging modality in oncology [1]. PET/MRI is expected to be more valuable than PET or CT, alone or combined, because PET/MRI involves a better contrast in soft tissues and a lower radiation dose from the MRI system [1]. The advantage of clinical PET/MR is rather to replace PET/CT + MR, and reduce the radiation dose in comparison with PET/CT. DWI, a widely used technology for analyzing the motion of water molecules (Brownian motion) as a noninvasive diagnostic tool of tissue biology [4] by dissecting the texture of a biologic tissue based on the motion of water molecules at a microscopic level, is also available with PET/MRI [2]. ADC represents DWI for determining a tumor’s cellularity [3,5]. The higher cellular tumor results in more restriction to water molecule motion which, as a result, gives lower ADC values and vice versa [4]. This means that the water molecule’s motion reflects the signal loss on DWI, due to different water permeabilities through the structures [2,6]. Previous studies have proved the inversely proportional correlation between ADC and tumor cellularity [7,8]. ADC also was found to be effective in primary tumor assessment, differentiating between benign and malignant neoplasms, staging, and monitoring at post-treatment follow-up [9,10]. ADC was also found to be useful in predicting therapy response in head and neck squamous cell carcinoma (HNSCC) patients [11].

Owing to their ability to quantify glucose metabolism in tumors, FDG uptake values from PET imaging play an essential role in head and neck imaging, [12,13], which may also reflect the tumor’s aggressiveness and the risk of metastasis spreading to surrounding structures [14,15]. SUV is the most used metric to estimate glucose metabolism, and it has shown promise for predicting the presence of metastatic lymph nodes at the original assessment, as well as survival and recurrence [16]. The metabolic parameters, TLG and MTV have emerged as new parameters that can measure the glucose metabolism activity of tumors and have been found to be more effective than SUV, because tumor contour is considered when using MTV and TLG [17]. SUVmax does not reflect the metabolic activity of the entire lesion, but measures the highest glucose metabolism in the target ROI [18]. While, MTV represents the volume of the ^18^F-FDG activity in the lesion and TLG represents the sum of the SUV within the lesion. Furthermore, glucose metabolic activity is positively correlated to tumor cellularity [19,20].

Therefore, our study aimed to investigate the correlation between FDG parameters and ADC values, and focused, in-depth, on finding out if there is a correlation between tumor metabolic activity and cellularity, represented by ADC and SUVmax, TLG, and MTV, as well as assessing the ability of these imaging parameters to determine tumor aggressiveness, by predicting lymph node involvement.

## 2. Materials and Methods

### 2.1. Patients and Demographics

The Clinical Center, Regional and Local Research Ethics Committee (CCRLREC), University of Pecs Doctoral School of Health Sciences, and Somogy Megyei Kaposi Mor Educational Hospital, Pecs, Hungary (Approval Number: IG/04866.000/2020) approved this retrospective study [11]. The informed consent requirement was waived and confirmed by the (CCRLREC), and all methods were carried out following the applicable guidelines and laws (Declaration of Helsinki). From May 2016 to June 2019, 109 patients with confirmed HNC had their disease staged and assessed by 18F-FDG PET/MRI. (1) Patients had to have untreated main HNC, (2) they had to have PET/CT and PET/MRI with DWI sequences, and (3) they had to be non-smokers, as well as a single tracer injection session. Exclusion criteria: (1) patients who had non-measurable ADC, or FDG parameters; (2) patients with motion artefact or bad image quality. Finally, a total of 71 patients were included in our study, see Table 1. Biopsy was the gold standard method for malignancy confirmation for all patients after PET/MRI examination.

### 2.2. PET/MRI Imaging

The work strategy and procedure have been published elsewhere [11,12,21]. In brief, the examinations were conducted in a dedicated PET/MRI (3 T) equipment (Biograph mMR, Siemens AG, Erlangen, Germany). Patients were instructed to fast for at least 6 h and had their blood sugar levels checked to guarantee euglycemia before receiving the 18F-FDG injection intravenously. PET/MRI was conducted in the supine posture, images were captured with head and neck coils. PET/MRI parameters were also included (ADC, SUV, TLG, and MTV). MRI sequences were T2-weighted TSE turbo inversion recovery magnitude (TIRM) (TR/TE/TI 3300/37/220 ms, FOV: 240 mm, slice thickness: 3 mm, 224 × 320) coronal plan, T1-weighted turbo spin-echo (TSE) (TR/TE 800/12 ms, FOV: 200 mm, slice thickness: 4 mm, 224 × 320), and T1-weighted TSE Dixon fat suppression (FS) (TR/TE 6500/85 ms, FOV: 200 mm, slice thickness: 4 mm, 256 × 320) transversal and were acquired without an intravenous contrast agent [11,12,21].

### 2.3. Image Analysis

**Figure 1:**
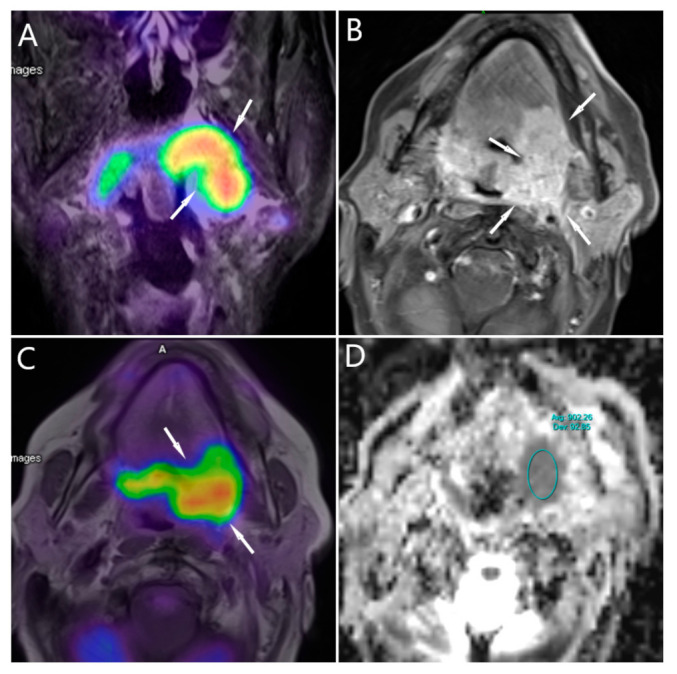
(**A**–**D**) and ^18^F-FDG measurements of 67 male patients with Oropharyngeal carcinoma. (**A**) T2-PET_tirm coronal MRI shows the intensive FDG accumulation (arrow). (**B**) T1-TSE-sagittal shows the extent of the tumor, lateral pharyngeal wall into the tongue root to the left tongue body (arrows). (**C**) T1-PET fused image shows the ROI within the tumor (arrows), and (**D**) DWI/ADC map showing the average and standard deviation of the ADC value.

All methods of image analysis were previously published [11,12,21]. In short, a fixed 2.5 threshold of SUV was used for tumor SUVmax for both MTV and TLG, as proposed by Pak et al. [13]. DWI measurements were previously mentioned [12,21]. ‘Avg’ represents the average ADC values for all voxels within the ROI and ‘Dev’ Represents the standard deviation, see Figure 1.

### 2.4. Statistical Analysis

SPSS 25 was used to conduct a statistical analysis (IBM SPSS Statistics, Armonk, NY, USA). For variables with a normal distribution, descriptive statistics (mean and standard deviation) were used; whereas, for variables with a non-normal distribution, median and interquartile ranges were used. The Spearman rank correlation (r) was used to estimate the association between ^18^F-FDG parameters and ADC values and tumor size (continuous variable). ANOVA or a Kruskal–Wallis test were performed on the clinicopathological features that may affect the 18F- FDG and ADC of the tumor. By combining variables with *p* < 0.1 in a univariate analysis, a multiple linear regression analysis was used to find those that were independently linked with imaging parameters. To transform statistically significant differences in the univariate analysis into the multivariate linear regression model, we used a transforming function to convert variables with non-normal distribution into a normal distribution, then the factors were added one by one (Stepwise). A Mann–Whitney test and independent-sample T-test were applied to the imaging parameters after the patients were grouped based on lymph node involvement into positive (N+) and negative lymph nodes (N−). A *p*-value <0.05 indicated a statistically significant result.

## 3. Results

**Figure 2:**
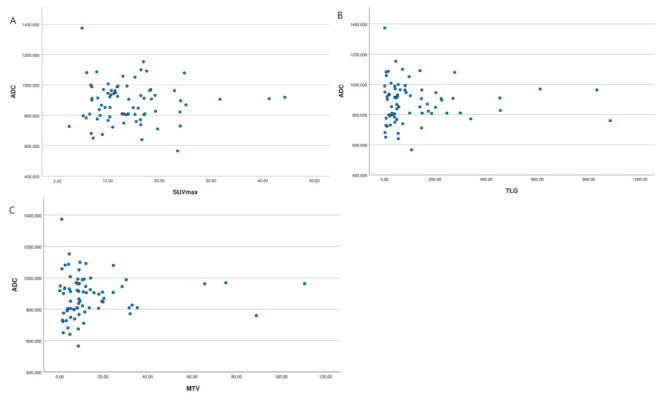
Scatter diagram showing the correlation between the ADCmean and (**A**) SUVmax, (**B**) TLG and (**C**) MTV. No significant linear correlation was observed between ADCmean and any of the 18F-FDG parameters, *p* > 0.05.

A summary of the measurements can be found in Supplementary file (Table S1). Spearman’s correlation coefficient was applied on ^18^F-FDG parameters and ADC values; the results show that ^18^F-FDG parameters (SUVmax, TLG and, MTV) were not correlated with ADC values (r = −0.184, *p* = 0.125, r = −0.182, *p* = 0.248, and r = −0.037, *p* = 0.756), respectively. A summary of correlations is shown in Table 2 and Figure 2A–C.

Moreover, the Spearman correlation coefficient was used to assess the correlation between 18F-FDG and tumor size (tumor size was measured as the maximum diameter of the tumor in pathologic results, mean size was 49.8 *±* 2.5 mm). The results show that 18F-FDG parameters (SUVmax, TLG and MTV) were significantly and positively correlated with tumor size (r = 0.456, *p* = 0.001; r = 0.794, *p* = 0.001; and r = 0.739, *p* = 0.001), respectively. ADC, on the other hand, showed no significant correlation with tumor size (r = −0.088, *p* = 0.464), see Table 2.

For a clinicopathological comparison, we compared primary tumor FDG (SUVmax, MTV, and TLG) and ADC parameters with sex, T stages, N stages, M stages (7th Edition American Joint Committee on Cancer pathological staging criteria), [14] localization, and the degree of differentiation (grades). The results show that N stages were correlated with higher SUVmax, (*p* = 0.023). T stages and N stages were correlated with TLG values (*p* = 0.006 and *p* = 0.033, respectively). T stages were correlated with MTV values (*p* = 0.001). Lower ADC, on the other hand, was found to be correlated with the degree of differentiation (*p* = 0.05), with a tendency for ADC to correlate with N stages, (*p* = 0.092). No other significant correlations were observed, (*p* > 0.05) for all parameters, see Table 3.

Multiple regression was recruited for factors that showed a statistically significant correlation in univariate analysis, to investigate the factor that influences the change in (SUVmax, TLG, MTV, and ADC). The results showed that tumor size and N stage were independent factors influencing SUVmax, (*p* = 0.001 and *p* = 0.008, respectively). Tumor size was an independent factor influencing TLG and MTV (*p* = 0.001 and *p* = 0.001, respectively). Tumor grade was found to be an independent influencing factor of ADC (*p* = 0.05). Table 4.

When removing the effect of the tumor size, SUVmax was correlated with N stages (*p* = 0.011), but not with T stages (*p* = 0.838); TLG was significantly correlated with both T stage and N stages (*p* = 0.018 and *p* = 0.034); and MTV was correlated to T stages (*p* = 0.001).

**Figure 3:**
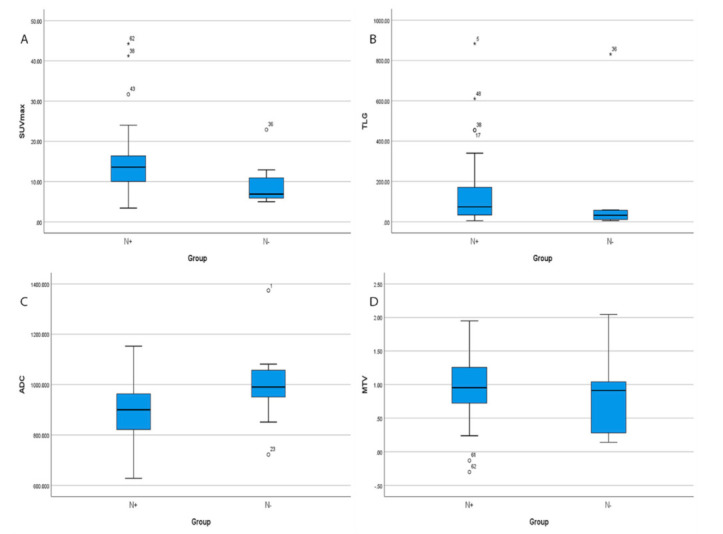
Boxplots displaying the distribution of SUVmax, TLG, ADC and MTV (**A**–**D**) according to lymph nodes status. (**A**) SUVmax values of positive lymph nodes tumors were significantly higher than those lymph nodes negative tumors (*p* = 0.004). (**B**) TLG show no significant difference between positive and negative lymph node (*p* = 0.084). (**C**) ADC values of positive lymph nodes tumors were not significantly between positive and negative lymph nodes (*p* = 0.074) and finally, (**D**) MTV positive lymph nodes tumors and negative lymph nodes tumors were not statistically significant difference (*p* = 0.342).

To investigate the ability of FDG and ADC parameters to predict lymph node involvement, we classified the patients based on lymph node involvement into negative and positive groups (N− and N+) and compared them using these parameters. Our results show that SUVmax revealed statistically significant differences (*p* = 0.004); while TLG, MTV and ADC did not (*p* > 0.05). Figure 3A–D.

## 4. Discussion

The present study demonstrated that PET/MR provides valuable imaging data for HNC patients. Various pathological factors were associated with PET/MR results and may have a role in the evaluation of the prognosis of patients with HNC. PET/MRI offers different imaging data for studying tumor microstructure environments. Previous data demonstrated an inverse correlation between ADC value, derived from DWI, and cellularity [3,4,5,8]. FDG imaging parameters, on the other hand, were found to be positively correlated with cellularity [15,19,20]. Although the glucose metabolism and cellularity of tissue are two different biological biomarkers of a tumor, an inverse correlation between 18F-FDG and DWI has been suggested [15]. This hypothesis was proposed because both 18F-FDG and ADC were correlated with tumor cellularity [16].

In our study, the results showed that FDG uptake parameters (SUVmax, TLG, and MTV) were not significantly correlated with the ADC values. Similarly to Min et al., who found no significant correlation between ADCmean with SUVmax and SUVmean, no significant correlation was found between ADCmean and both MTV and TLG [22]. Surov et al., in a recent study, reported no significant correlation between ADCmean and SUVmax or SUVmean [23], and others [24,25,26,27].

On the other hand, contrarily to our results, Nunez et al. observed, in their study of HNSCC, a significant inverse correlation between the SUV and the ADC [27]. Nakajo et al. also observed that SUVmax was correlated inversely with ADCmean [28]. Han et al. reported a slightly significant inverse correlation between SUV and ADC. They also found a negative significant correlation between ADC and TLG [29].

Our explanation for the lack of correlation is the fact that both imaging parameters explain different tissue microstructures characteristics, where DWI assesses the water molecule motion in the tissue and is affected by the cellularity, proliferation rate, and cell counts, which in clinical use are affected by ROI size placement and interobserver variability [30]. On the other hand, metabolic activity was found to be independent of tumor size and shape, because the tumor is segmented by adaptive thresholding [16].

Furthermore, the tumor’s clinicopathological characteristics were correlated to the imaging parameters, and the results reveal different correlations, as such; the primary tumor SUVmax was significantly correlated with the N stages; higher values of SUVmax were found in patients with a higher N stage. According to Zheng et al., there was a positive significant correlation between lymph nodes status and SUVmax; a higher SUVmax resulted in more lymph node metastasis, which means that SUVmax has a predictive role in lymph node diagnosis [31]. Micco et al. reported a significant correlation between lymph node occurrence and SUVmax and TLG [32]. Morand et al. observed similar results, where higher lymph node involvement was found in patients with higher primary tumor SUVmax [33]. In the same study, the authors reported that TLG did not correlate with lymph node status [33]. In our study, no significant correlation was observed between MTV and lymph node status, a similar result was reported by Morand et al. [33] and Chan et al. [34]. In summary, N stages and tumor size were independent factors influencing SUVmax. Tumor size and tumor T stages were independent factors influencing TLG and MTV. Thus, SUVmax might be a promising imaging biomarker to predict tumor aggressiveness.

ADC, on the other hand, shows a significant correlation with the tumor degree of differentiation, this results from the fact that a higher-grade tumor (G3) shows more restriction to water molecules, which as a result decreases the ADC value. Additionally, ADC did not show any significant correlation with T stages, N stages, or tumor size. Similar results were found by Nakajo et al. [28]. Moreover, other authors revealed the same findings [35,36]. In contrast, Abdel Razek et al., in their study of Nasopharyngeal carcinoma have reported a statistically significant difference between primary tumor ADC and nodal involvement [4]. While in our study, the explanation of different results was due to the heterogeneity of the patient’s sample, which contained multiple primary tumor localization, and, thus, different anatomical and histological components were involved.

Although several studies have investigated the diagnostical role of 18F-FDG and ADC for determining tumor aggressiveness in different cancers [32,37,38,39,40,41,42], none of the studies have compared the efficacy of different PET/MRI imaging biomarkers in HNC tumor aggressiveness prediction. Thus, to our knowledge, this is the first study to compare PET/MRI system-derived imaging parameters in lymph node involvement in HNC. Our results show that SUVmax was found to be able to differentiate between the two lymph node groups (N+ and N−) based on the primary tumor measurements, which as a result might help to predict tumor development and prognosis. The importance of the successful prediction of tumor aggressiveness and lymph node involvement might help in daily practice, to increase the effectiveness of the therapy.

Based on our study results and findings, there were several correlations between PET/MRI imaging parameters and clinical tumor characteristics, and we suggest that glucose metabolism assessed by 18F-FDG and cellularity assessed by ADC have different roles in cancer evaluation; therefore, we recommend PET/MRI as a combined examination, rather than PET or MRI alone.

As for this study’s limitations, first is the heterogeneity of the tumor localization. Second, our study focused on the search for a correlation between 18F-FDG, ADC, and histopathological features only in HNC. Third, associations with other functional tumor parameters such as apoptosis factors were not analyzed. Fourth, the design of the study was retrospective.

## 5. Conclusions

Our results revealed no linear correlation between FDG PET and ADC MR parameters. FDG PET-based glucose metabolic and DWI MR derived cellularity data may represent different biological aspects of HNC tumors, and simultaneous PET/MR imaging could provide complementary diagnostic information. SUVmax showed a higher accuracy in predicting tumor aggressiveness than DWI.

## Figures and Tables

**Table 1:** Patient demographics.

Number of Patients	71
Mean Age (y)	(61.6 ± 0.8)
Men	49 (69.0%)
Women	22 (31.0%)
Histologic Grade	
Grade 1	12 (16.9%)
Grade 2	41 (57.7%)
Grade 3	18 (20.4%)
Localization	
Pharyngeal	32 (45.1%)
Laryngeal	15 (21.1%)
Oral	22 (33.8%)
T category	
T1	4 (5.6%)
T2	19 (26.8%)
T3	26 (36.6%)
T4	22 (31.0%)
N category	
N0	10 (14.1%)
N1	9 (12.7%)
N2	45(63.4%)
N3	7 (9.9%)
M Category	
M0	63 (88.7%)
M1	8 (11.3%)
N groups	
N+	61 (85.9%)
N−	10 (14.1%)

**Table 2:** Summary of correlations between FDG and DWI imaging parameters.

Parameter		ADC	SUVmax	TLG	MTV	Tumor Size
ADC	Spearman (rho)		−0.184	−0.182	−0.037	−0.088
	Sig. (2-tailed)		−0.125	0.129	0.756	0.464
SUVmax	Spearman (rho)					0.456 *
	Sig. (2-tailed)					**0.000**
TLG	Spearman (rho)					0.794 *
	Sig. (2-tailed)					**0.000**
MTV	Spearman (rho)					0.739 *
	Sig. (2-tailed)					**0.000**

* Significant at a level of 0.05, significant result in bold.

**Table 3:** Clinicopathological comparison with FDG and DWI imaging parameters.

Grouping	SUVmax	TLG	MTV	ADC
SEX	*p* = 0.314	*p* = 0.522	*p* = 0.784	*p* = 0.897
T stages	*p* = 0.267	***p =*** **0.006**	***p*** **= 0.001**	*p* = 0.880
N stages	***p =*** **0.023**	***p =*** **0.033**	*p* = 0.605	*p* = 0.092
M stages	*p* = 0.283	*p* = 0.785	*p* = 0.913	*p* = 0.347
Grades	*p* = 0.233	*p* = 0.310	*p* = 0.713	***p =*** **0.050**
Localization	*p* = 0.389	*p* = 0.128	*p* = 0.367	*p* = 0.270

Kruskal–Wallis for multi-categorical variables (T stages, N stages, localization, and tumor grades) and Mann–Whitney test for two categorical variables (sex, M stages) were used with (SUVmax, TLG and MTV). ANOVA and independent sample *t*-test were used with ADC values. Significant results are highlighted in bold.

**Table 4:** Multiple Regression Analysis Showing the Effects of Prognostic Factors on 18f-FDG parameters.

Prognostic Factors	B	T	*p* Value
SUVmax			
Tumor size	0.409	3.333	0.001 *
T stages	N/A	N/A	N/A
N stages	0.227	1.995	0.022 *
TLG			
Tumor size	0.767	8.988	0.000 *
T stages	−0.050	−0.598	0.552
N stages	0.119	1.500	0.138
MTV			
Tumor size	0.662	6.857	0.000 *
T stages	0.140	1.473	0.146
N stages	N/A	N/A	N/A
ADC			
N stages	0.043	2.042	0.069
Tumor grades	−0.021	−1.846	0.045 *

* Significant result; N/A: Not assessed. Significant results are highlighted in bold.

## Data Availability

The datasets used and/or analyzed during the current study are available from the corresponding author on reasonable request.

## Supplementary Materials

The following supporting information can be downloaded at: https://www.mdpi.com/article/10.3390/cancers14030847/s1, Table S1: Summary of measurements.

## Abbreviations

PET/MRI	Positron Emission Tomography/Magnetic Resonance Imaging
SUV	Standardized Uptake Value
MTV	Metabolic Tumor Volume
TLG	Metabolic Tumor Volume
CRT	Chemo-Radiotherapy
HCC	Head and Neck Cancer
FDG	Fluorodeoxyglucose
AJCC	American Joint Committee on Cancer
(G)	Grade
