# Supplementary material for: Correlation between Tissue Cellularity and Metabolism Represented by Diffusion-Weighted Imaging (DWI) and 18F-FDG PET/MRI in Head and Neck Cancer (HNC)
Source: Cancers (Basel). 2022 Feb 8;14(3):847. doi: 10.3390/cancers14030847 (PMC8833888; doi:10.3390/cancers14030847)
Supplement: Supplementary file 1 [file cancers-14-00847-s001.zip › cancers-1574343-supplementary.pdf]

Table S1. Summary of measurements.

| Parameters | ADCmean ($10^{-3}mm^2/s$) | SUVmax | TLG | MTV |
|---|---|---|---|---|
| Sex | | | | |
| Male | 0.906±0.44 | 11.06±0.55 | 125.9±1.56 | 13.87±1.61 |
| Female | 0.910±0.85 | 9.48±0.39 | 128.6±2.05 | 17.76±2.59 |
| T stages | | | | |
| T1 | 0.934±0.18 | 6.24±0.32 | 17.66±0.66 | 2.68±0.18 |
| T2 | 0.893±0.30 | 10.30±0.45 | 74.58±1.02 | 6.90±0.53 |
| T3 | 0.900±0.23 | 11.96±0.62 | 142.1±1.13 | 16.01±1.36 |
| T4 | 0.924±0.49 | 9.96±0.39 | 173.4±2.42 | 22.70±0.29 |
| N stage | | | | |
| N0 | 0.960±0.12 | 7.60±0.52 | 130.7±2.83 | 19.8±0.35 |
| N1 | 0.905±0.10 | 9.38±0.38 | 45.36±0.31 | 10.6±0.88 |
| N2 | 0.909±0.11 | 11.54±0.57 | 163.9±2.53 | 16.6±0.19 |
| N3 | 0.889±0.16 | 10.95±0.14 | 226.7±3.31 | 24.6±0.33 |
| M stage | | | | |
| M0 | 0.913±0.13 | 10.36±0.50 | 119.9±1.52 | 14.64±0.18 |
| M1 | 0.866±0.91 | 12.26±0.61 | 180.3±2.91 | 18.45±2.87 |
| Localization | | | | |
| Pharyngeal | 0.822±0.24 | 10.81±0.53 | 156.4±1.82 | 17.19±1.81 |
| Laryngeal | 0.946±0.28 | 11.78±0.56 | 88.35±0.78 | 9.14±0.93 |
| Oral | 0.917±0.49 | 9.59±0.43 | 111.1±1.96 | 15.86±2.11 |
| Grade | | | | |
| G1 | 0.978±0.24 | 9.09±0.51 | 164.8±2.54 | 28.2±0.36 |
| G2 | 0.907±0.27 | 10.86±0.42 | 120.1±1.24 | 13.7±0.14 |
| G3 | 0.874±0.33 | 11.10±0.71 | 197.2±3.87 | 17.7±0.22 |
| Overall | 0.907±0.81 | 10.57±0.53 | 126.9±1.17 | 15.07±0.22 |
